# Three-dimensional spatial analysis of missense variants in *RTEL1* identifies pathogenic variants in patients with Familial Interstitial Pneumonia

**DOI:** 10.1186/s12859-018-2010-z

**Published:** 2018-01-23

**Authors:** R. Michael Sivley, Jonathan H. Sheehan, Jonathan A. Kropski, Joy Cogan, Timothy S. Blackwell, John A. Phillips, William S. Bush, Jens Meiler, John A. Capra

**Affiliations:** 1 Department of Biomedical Informatics, Vanderbilt University, Nashville, USA; 2 Department of Biochemistry and Center for Structural Biology, Vanderbilt University, Nashville, USA; 3 Department of Medicine, Vanderbilt University, Nashville, USA; 4 Department of Pediatrics, Vanderbilt University, Nashville, USA; 5 Department of Quantitative and Population Health Sciences, Case Western Reserve University, Cleveland, OH 44106 USA; 6 Department of Chemistry and Center for Structural Biology, Vanderbilt University, Nashville, USA; 7 Department of Biological Sciences, Vanderbilt Genetics Institute, and Center for Structural Biology, Vanderbilt University, Nashville, USA

## Abstract

**Background:**

Next-generation sequencing of individuals with genetic diseases often detects candidate rare variants in numerous genes, but determining which are causal remains challenging. We hypothesized that the spatial distribution of missense variants in protein structures contains information about function and pathogenicity that can help prioritize variants of unknown significance (VUS) and elucidate the structural mechanisms leading to disease.

**Results:**

To illustrate this approach in a clinical application, we analyzed 13 candidate missense variants in regulator of telomere elongation helicase 1 (*RTEL1*) identified in patients with Familial Interstitial Pneumonia (FIP). We curated pathogenic and neutral *RTEL1* variants from the literature and public databases. We then used homology modeling to construct a 3D structural model of RTEL1 and mapped known variants into this structure. We next developed a pathogenicity prediction algorithm based on proximity to known disease causing and neutral variants and evaluated its performance with leave-one-out cross-validation. We further validated our predictions with segregation analyses, telomere lengths, and mutagenesis data from the homologous XPD protein. Our algorithm for classifying *RTEL1* VUS based on spatial proximity to pathogenic and neutral variation accurately distinguished 7 known pathogenic from 29 neutral variants (ROC AUC = 0.85) in the N-terminal domains of RTEL1. Pathogenic proximity scores were also significantly correlated with effects on ATPase activity (Pearson *r* = −0.65, *p* = 0.0004) in XPD, a related helicase. Applying the algorithm to 13 VUS identified from sequencing of *RTEL1* from patients predicted five out of six disease-segregating VUS to be pathogenic. We provide structural hypotheses regarding how these mutations may disrupt RTEL1 ATPase and helicase function.

**Conclusions:**

Spatial analysis of missense variation accurately classified candidate VUS in *RTEL1* and suggests how such variants cause disease. Incorporating spatial proximity analyses into other pathogenicity prediction tools may improve accuracy for other genes and genetic diseases.

**Electronic supplementary material:**

The online version of this article (doi: 10.1186/s12859-018-2010-z) contains supplementary material, which is available to authorized users.

## Background

The use of next-generation sequencing to study families with pulmonary diseases has led to the identification of novel genes and mechanisms associated with the inherited forms of pulmonary arterial hypertension [1–5] and pulmonary fibrosis [6–8]. Genetic variation in telomere-related genes is the predominant cause of pulmonary disease (when genetic etiology is known). Even when the genetic cause is unknown, such as with idiopathic pulmonary fibrosis, telomere shortening in peripheral blood mononuclear cells [9–11] and type II alveolar epithelial cells [6, 11] is commonly observed in patients and families. The mechanism through which telomere dysfunction leads to lung fibrosis is not clear, but may involve premature senescence of progenitor cells in the distal lung [12–14]. Among families with pulmonary fibrosis (Familial Interstitial Pneumonia, FIP), whole exome sequencing (WES) studies have identified that variation in a few genes is responsible for disease risk. The most commonly mutated genes in FIP patients are *TERT* (10–15% of cases) [15, 16], *RTEL1,* and *PARN* (3–4% of cases each) [6, 7]. Most FIP mutations identified to date are very rare or novel. Rare variation presents challenges when using genetic information in clinical practice, since most newly identified variants in FIP-associated genes are considered variants of unknown significance (VUS).

Predicting the effects of rare missense VUS on protein function is particularly challenging; some variants are tolerated while others lead to dramatic alterations in protein structure, trafficking/localization, or function [17]. Classical genetic approaches, including linkage analysis, are often limited by small family size, disease onset late in life, and in the case of telomere-related genes such as *RTEL1*, may also be confounded by the inheritance of short telomeres (and thus increased disease risk) without inheritance of the causal allele. Assigning pathogenicity to VUS has important implications for genetic testing and family counseling, and may soon impact treatment decisions. While functional testing of variants remains the gold standard, in many cases this is not feasible in a sufficiently timely manner to impact clinical care. Numerous *in-silico* algorithms have been developed to predict VUS pathogenicity by analyzing evolutionary conservation patterns and/or biochemical characteristics of amino-acid substitutions (e.g., SIFT [18], PolyPhen [19], VAAST [20], GERP [21], CADD [22], VIPUR [23]). However, these methods frequently present discordant classifications [20] and rarely provide specific mechanistic hypotheses about the functional effects of VUS. Novel approaches are required that incorporate RTEL1-specific information to improve pathogenicity prediction.

We screened FIP families from our registry for rare variants in *RTEL1* and identified 13 rare missense VUS. We hypothesized that pathogenic *RTEL1* variants likely affect critical functions and/or protein interactions and thus would co-localize in three-dimensional space. To test this hypothesis, we used homology modeling to predict the tertiary structure of RTEL1 and identified a spatial cluster of variants with known disease-association in RTEL1’s helicase domains. We then developed an algorithm to classify missense VUS based on their spatial proximity to known pathogenic and neutral variants with the expectation that VUS near the pathogenic cluster are more likely contribute to disease. The approach outperformed two common pathogenicity prediction methods in cross-validation and predicted the pathogenicity of disease-segregating VUS with high accuracy. Our study supports the likely pathogenicity of novel FIP-associated rare variants, generates a new homology model of RTEL1’s 3D structure, supports quantitative spatial analysis in protein structure as a powerful approach to classify VUS in *RTEL1,* and suggests this technique may have broad applicability to other genes and genetic diseases.

## Methods

### Subjects and samples

We trained our spatial proximity prediction algorithm using putatively neutral *RTEL1* missense variants from the 1000 Genomes Project [24] that were not otherwise associated with disease and pathogenic missense variants causing severe pediatric, autosomal recessive Hoyeraal-Hreidarsson syndrome collected from previous literature [25–31]. We evaluated the performance of our prediction algorithm using rare missense variants of unknown significance from patients with Familial Interstitial Pneumonia (FIP). Subjects were identified from the Familial Interstitial Pneumonia (FIP)/Familial Pulmonary Fibrosis (FPF) registries at Vanderbilt University, the University of Colorado, and National Jewish Hospital [6]. FIP was defined by the presence of Idiopathic Interstitial Pneumonia (IIP) in two or more family members, including interstitial pulmonary fibrosis (IPF) in at least one individual. Phenotypes of subjects selected for sequencing were ascertained using ATS/ERS criteria for IIP [32]. The affected status of deceased individuals was determined by review of available medical records, autopsy material, or by death certificates. DNA was isolated from blood and/or paraffin-embedded lung tissue using a PureGene Kit (Gentra Systems, Minneapolis, MN). Rare missense variants (MAF < 0.001) in *RTEL1* were curated from whole-exome sequencing data as previously reported [6] (*n* = 189 families) or targeted modified Sanger sequencing of *RTEL1* (*n* = 184 families) (Additional file 1: Figure S1). Co-segregation and telomere length measurements were performed as previously described [6]. VUS co-segregation with disease and short telomeres were considered evidence for pathogenicity and represent true-positives in our analysis.

### Protein structural analysis

We quantified the spatial proximity of each VUS to each known pathogenic and neutral variants using the NeighborWeight transformation of the 3D Euclidean distance between the centroid of each amino acid side chain [33],

$$ NeighborWeight\left(x,y, lower bound, upper bound\right)=\left\{\begin{array}{c}\begin{array}{c}1, if\ {d}_{x,y}\le lower bound\\ {}\frac{1}{2}\left[\cos \left(\frac{d_{x,y}- lower bound}{upper bound- lower bound}\times \pi \right)+1\right],\\ {} if lower bound<{d}_{x,y}< upper bound\end{array}\\ {}0, if\ {d}_{x,y}\ge upper bound\end{array}\right. $$

where *d*_*x*, *y*_ is the distance between VUS *x* and variant *y* from set *Y* (pathogenic or neutral) and the bounds give upper and lower bounds in angstroms. This transformation up-weights the contribution of nearby variants and down-weights distant variants that are less likely to have similar functional effects (Additional file 1: Figure S3). To capture neighboring residues with the potential for direct interaction, the lower bound was set to 8 Å. The upper bound was set to 24 Å to capture variants potentially impacting the same functional domain or element. We then calculated the proximity *P* of each VUS *x* to variants in dataset *Y* using the weighted-average of transformed distances,

$$ {P}_{x,Y}=\sum \limits_y^Y\frac{NeighorWeight\left(x,y,8,24\right)}{\left|Y\right|} $$

To classify VUS, we calculated the difference in the pathogenic and neutral proximity scores,

$$ \Delta  {P}_x={P}_{x, pathogenic}-{P}_{x, neutral} $$

such that candidate VUS in closer proximity to pathogenic variation than neutral variation receive positives scores. We refer to *∆P* as the pathogenic proximity score.

We evaluated the predictive power of the pathogenic proximity score using leave-one-out cross-validation on the known pathogenic and neutral variants [34]; each variant was predicted to be pathogenic or neutral by its proximity to all other variants. We quantified the performance of each prediction method using the area under the receiver operating characteristic curve (ROC AUC). The ROC curve plots true positive rate, the proportion of true positives (pathogenic variants) predicted to be positive, versus false positive rate, the proportion of true negatives (neutral variants) predicted to be positive, as a function of prediction rank. The ROC AUC is equivalent to the probability that a randomly selected positive is ranked higher than a randomly selected negative; thus, perfect separation of positives and negatives produces a ROC AUC of 1.0 and random ordering produces a ROC AUC of 0.5. We compared the performance of the pathogenic proximity score with other pathogenicity prediction methods, including ConSurf evolutionary conservation scores [35], SIFT [18], and PolyPhen2 [19]. A brief description of each approach is provided in the Additional file 1: Supplemental Methods.

## Results

### Constructing a structural model of RTEL1

The protein structure for RTEL1 has not yet been experimentally determined, so we constructed a computationally derived homology model. To begin, we applied nine computational modeling algorithms to the protein sequence: GeneSilico [36], HHpred [37], I-TASSER [38], M4T [39], Pcons5 [40], Phyre2 [41], RaptorX [42], Robetta [43], and SWISS-MODEL [44]. RaptorX produced the highest-coverage model, which consisted of two well-folded domains spanning residues 1–769 and 881–1151. This model was based on seven PDB structures: 4a15 [45], 3crv [46], 2fi7 [47], 2gm7 [48], 4pjq [49], 2vrw [50], 4a64 [51]. To improve quality, the model was relaxed using Rosetta version 2015.19 [52], and then subjected to 1000 rounds of loop_modeling [53] using perturb_kic_with_fragments. This new structural model of RTEL1 is available as Additional file 2.

### Known pathogenic missense variants in RTEL1 cluster in 3D structure

**Fig. 1:**
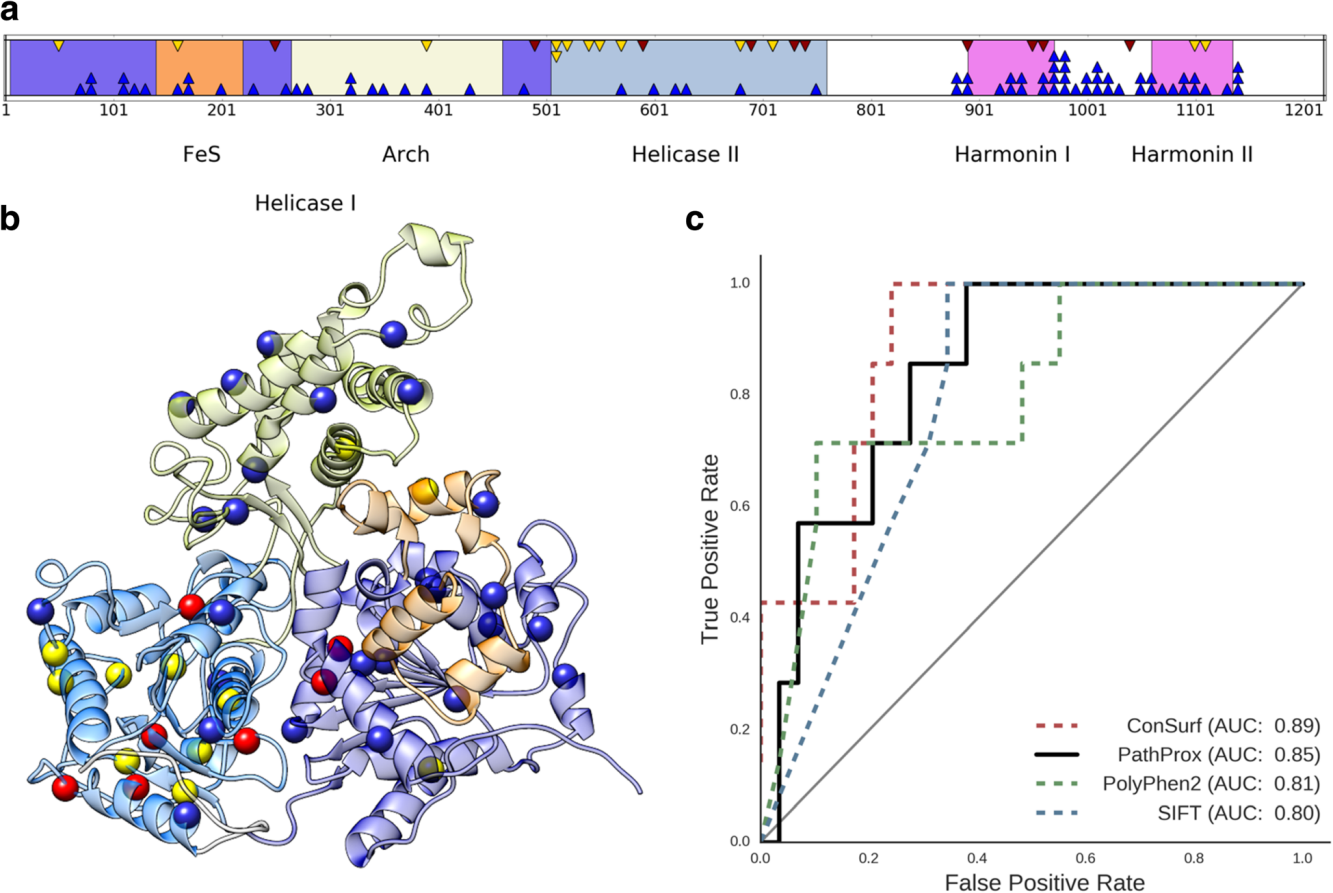
Identification and classification of novel pathogenic FIP variants in RTEL1. **a** The locations of known pathogenic (red), putatively neutral 1000 Genomes (blue), and FIP VUS (yellow) missense variants are plotted in the context of the RTEL1 protein sequence and known domains. **b** The locations of pathogenic, putatively neutral, and candidate variants in the RTEL1 N-terminal structural model. **c** Leave-one-out cross validation of the pathogenic proximity score applied to characterized *RTEL1* variants yielded an improved area under the ROC curve (AUC) relative to PolyPhen2 and SIFT, but was outperformed by evolutionary conservation scores. These results demonstrate that considering the 3D spatial distribution of known pathogenic and neutral variants can identify pathogenic hotspots and assist in the classification of VUS

To analyze the 3D distribution of disease-associated RVs in *RTEL1*, we mapped known pathogenic and neutral variants onto the sequence and structure of RTEL1 (Fig. 1). Because the relative orientation of the N- and C-terminal models (residues 1–769 and 881–1151) is unknown, we analyzed variants in these models separately. There were relatively few candidate VUS in the smaller C-terminal model, so we focused further analyses on the N-terminal model. Details of the C-terminal analysis are described in the Additional file 1: Supplemental Results (Table S3 and Figure S4). In the N-terminal model, we observed spatial clustering of pathogenic variants in helicase domain II (Fig. 1a) and near the structural interface of helicase domains I and II (Fig. 1b). This tendency was not observed among neutral variants, which were distributed throughout the protein structure. The distinct spatial distributions of pathogenic and neutral variation suggest that clustering is characteristic of pathogenic variation in *RTEL1* and that disease-causing missense RVs in *RTEL1* disrupt similar protein functions.

### Spatial proximity analysis accurately classifies pathogenic and neutral RTEL1 variants

Based on the observed differences between neutral and pathogenic variant distributions, we hypothesized that candidate VUS could be classified by their relative spatial proximity to known pathogenic and neutral variants. To evaluate this, we used leave-one-out cross-validation to calculate pathogenic proximity scores (Δ*P*) for each known pathogenic and neutral variant in the N-terminal model of RTEL1 (Table S1) and then plotted ROC and PR curves to measure how accurately the proximity score predicts pathogenicity. Classifying variants by their pathogenic proximity score performed well (Fig. 1c); the approach yielded a ROC AUC of 0.85.

To estimate the sensitivity of the proximity-based prediction method to the number of known pathogenic variants, we recomputed pathogenic proximity scores using all possible subsets of pathogenic variants and then calculated the ROC and PR AUC for each subset (Additional file 1: Figure S1). As expected, performance increases as the number of known pathogenic mutations considered increases; the mean ROC AUC is 0.62 when only two pathogenic variants are known and 0.82 when six variants are considered. This suggests that performance will increase as more pathogenic variants are identified. However, we caution that the number of known pathogenic variants required will likely vary substantially based on the structure and function of the protein of interest.

We then compared the performance of our pathogenic proximity score to a representative set of current methods for in silico pathogenicity prediction: ConSurf evolutionary conservation [35], SIFT [18], PolyPhen2 [19] (Fig. 1c). The pathogenic proximity score outperformed PolyPhen2 (ROC AUC = 0.81) and SIFT (ROC AUC = 0.80); evolutionary conservation had the best performance (ROC AUC = 0.89). The competitive ROC AUC with current methods and the relatively strong performance obtained with small numbers of known pathogenic variants demonstrates the predictive potential of spatial statistics, which are not currently used for variant pathogenicity prediction.

### The pathogenic proximity score identifies nearly all disease-segregating VUS as pathogenic

Given the predictive potential of the pathogenic proximity score, we applied our methodology to the 13 missense VUS identified from our FIP registry; six that segregate with disease, five that do not segregate with disease, and two for which segregation data was unavailable. The pathogenic proximity score classified eight VUS as deleterious (Table 1), including five VUS (V516 L, S540A, F559I, S688C, D719G) that co-segregated with disease and were found in subjects with short telomeres in peripheral blood mononuclear cells, a biomarker of reduced RTEL1 activity [9–11] (Additional file 1: Figure S2). Two false positives (A528E, R574W) did not co-segregate with disease or were found in subjects with normal length telomeres. The VUS receiving the highest pathogenic proximity score was the uncharacterized W512C variant; there was not sufficient DNA for telomere length measurement or DNA available from other affected individuals in this family for co-segregation analysis. Of the five VUS predicted to be neutral by the pathogenic proximity score, four (H161Q, Q397E, P1107L, F1110 L) did not co-segregate with disease. For comparison, no prediction method correctly classified all segregating variants, all prediction methods misclassified the two false positives, and only evolutionary conservation correctly classified the single false negative. Detailed structural hypotheses for the pathogenicity of W512C and the disease co-segregating VUS are provided in the Discussion.

**Table 1:** Pathogenicity predictions for RTEL1 missense VUS from FIP patients

Pos	Ref	Alt	Telomere %	Segregation	PPH2	SIFT	ConSurf	PathProx	Model
55	T	S	3%	Seg	0.00	1.00	**−0.56**	−0.02	N-terminal
516	V	L	1%	Seg	0.05	0.62	**−0.15**	**0.41**	N-terminal
540	S	A	2%	Seg	**0.57**	0.09	**−0.80**	**0.21**	N-terminal
559	F	I	6%	Seg	**1.00**	**0.00**	**−1.11**	**0.44**	N-terminal
688	S	C	1%	Seg	**0.91**	0.14	**−0.62**	**0.27**	N-terminal
719	D	G	8%	Seg	0.03	0.22	0.21	**0.05**	N-terminal
512	W	C	Unknown	Unknown	0.17	0.48	0.31	**0.47**	N-terminal
161	H	Q	Unknown	NonSeg	0.40	0.16	**−0.35**	−0.13	N-terminal
397	Q	E	94%	NonSeg	0.08	0.20	0.40	−0.09	N-terminal
528	A	E	58%	Unknown	**0.62**	**0.05**	**−0.75**	**0.08**	N-terminal
574	R	W	45%	NonSeg	**0.95**	**0.00**	**−0.53**	**0.07**	N-terminal
1107	P	L	6%	NonSeg	0.63	**0.01**		−0.13	C-terminal
1110	F	L	Unknown	NonSeg	0	1		−0.17	C-terminal

Variants are grouped by evidence for pathogenicity, which is inferred from disease co-segregation and patient telomere lengths. Variants that segregate with disease and short telomeres are treated as pathogenic (Additional file 1: Figure S1). Scores in bold indicate deleterious predictions. All thresholds were applied as recommended by each method

### RTEL1 pathogenic proximity scores correlate with decreased ATPase activity in XPD mutants

**Fig. 2:**
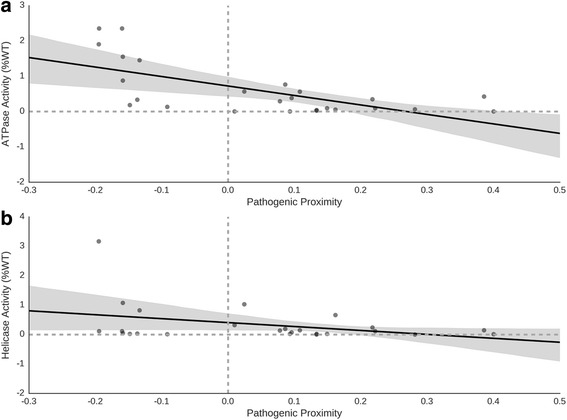
Pathogenic proximity scores in RTEL1 are correlated with decreased ATPase activity in mutagenesis studies of the homologous XPD protein. Pathogenic proximity scores were calculated for each missense mutation (*N* = 25) using their position relative to known pathogenic and neutral missense variants in RTEL1. **a** Pathogenic proximity was significantly correlated with a decrease in ATPase activity (Pearson *r* = −0.65, *p* = 0.0004), but **b** not significantly correlated with changes in helicase activity (Pearson *r* = −0.36, *p* = 0.08) in the homologous XPD protein

RTEL1 is a RAD3-related helicase in the DEAH subfamily of the Superfamily 2 (SF2) helicases and many FIP-associated variants in RTEL1 occupy domains that are highly conserved among proteins in this family [54]. To explore the mechanistic basis for the association of RTEL1 mutations with disease, we mapped mutagenesis data from two studies of the homologous protein, XPD, onto our human model of RTEL1 (Additional file 1: Figure S5; *N* = 15 Fan et al.; *N* = 9 Kuper et al., Additional file 3) [45, 46]. Spatial proximity to pathogenic variants in RTEL1 was significantly correlated with decreased ATPase activity (Pearson *r* = −0.65, *p* = 0.0004, Fig. 2a), but not with helicase activity (Pearson *r* = −0.36, *p* = 0.08, Fig. 2b). This suggests that pathogenic mutations in RTEL1 may perturb ATPase activity in a manner that leads to disease. Further detailed molecular hypotheses about how the individual segregating missense variants disrupt the structure and function of RTEL1—e.g., by disrupting protein-protein interactions (W512C) or DNA binding (F559I)—are provided in the Discussion.

## Discussion

Genetic variation in *RTEL1* is a common cause of FIP in families with known genetic etiology. Most disease-causing *RTEL1* variants are private or very rare mutations and appear to reduce RTEL1 levels and/or activity [6, 26]. Determining the pathogenicity of newly identified candidate VUS, particularly missense variants, presents a significant challenge in the diagnosis and treatment of patients and their family members that may be at risk [55]. A number of algorithms provide predictions for missense pathogenicity, but disagreement between algorithms is frequent; in one report, the correlation between SIFT and PolyPhen2 scores was only 0.4 [20]. Missense RVs in *RTEL1* are potentially actionable, so improved approaches to predicting pathogenicity could have a substantial clinical impact. In this report, we describe a novel, quantitative structural approach to predicting VUS pathogenicity, applied to 13 rare missense VUS in *RTEL1*.

We constructed a homology model of the structure of RTEL1 and analyzed missense VUS relative to the spatial distribution of known pathogenic and neutral variation. Five of six VUS that segregated with FIP in families were predicted to be pathogenic by our method, as well as one VUS without disease co-segregation or telomere length data. Below, we outline potential structural mechanisms of action – ranging from disruption of protein-protein or protein-DNA interactions to destabilization of the tertiary structure of the protein – for each segregating VUS.

### W512C

**Fig. 3:**
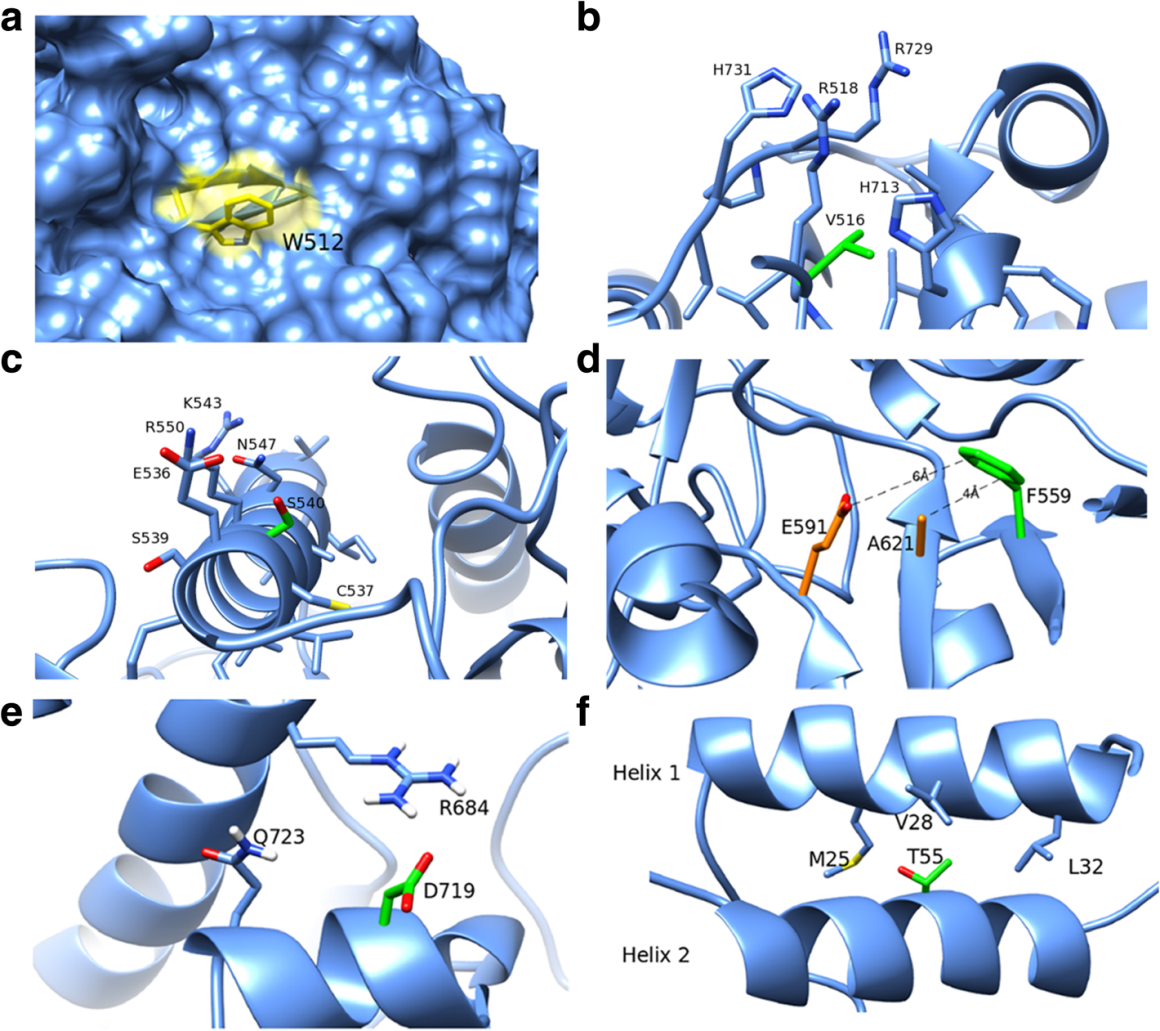
Structural hypotheses about the effects of six segregating *RTEL1* VUS. **a** W512 is predicted to lie on the surface of the protein. A mutation to cysteine has the potential to interfere with functionally important protein-protein interactions. **b** V516 forms a small well-packed hydrophobic core, which lies under a patch of positively charged surface residues. Mutation to leucine adds steric bulk and may induce structural rearrangements that disrupt DNA binding. **c** S540 is a polar residue predicted to lie on a surface-exposed alpha helix in the helicase II domain. Mutation to alanine may alter surface charge or cause rotation of the alpha helix. **d** F559 is buried in the core of the protein, in close proximity to residues predicted to form part of the DNA-binding cavity, including A621 and E591. Mutation to isoleucine removes steric bulk and is likely to leave a void in the hydrophobic core of the protein, disrupting structure and reducing stability. **e** D719 is predicted to fall in a surface-exposed helix. Mutation to glycine drastically reduces both the bulk and charge of the protein’s surface, and likely disrupts the helix at that point. **f** T55 is predicted to form part of the interface between helices 1 and 2 in RTEL1. Mutation to a serine would reduce the steric bulk and alter the packing between the two helices

W512 is a bulky aromatic residue found on the surface of the structural model (Fig. 3a). Surface-exposed aromatic side-chains are uncommon, and are often found to be important anchors for protein-protein binding surfaces. Replacing the tryptophan sidechain with the smaller, less hydrophobic cysteine may alter the shape and physicochemical character of a critical protein-binding surface of RTEL1, compromising its ability to perform its normal physiological function. This hypothesis is bolstered by the observation that this variant is ranked highest by our proximity score, indicating that other mutations found in close proximity to W512C – i.e. on or adjacent to the surface and likely to act through a common mechanism – are disease-linked. The importance of protein-protein interactions to RTEL1 function is underscored by the 46 unique interactions reported by the BioGrid database [56].

### V516-L

V516 is a moderately conserved, hydrophobic residue buried in the interior of the helicase II domain. It forms a small well-packed hydrophobic core, which lies under a patch of positively charged surface residues (R518, H713, R729, H731). Insertion of a leucine residue in this position is predicted to be destabilizing because of the additional steric bulk. Moreover, the structural rearrangement could disrupt the conformation of the basic surface patch, presumably affecting interaction with DNA.

### S540A

S540 is a polar residue predicted to lie on a surface-exposed alpha helix in the helicase II domain. Mutation of the hydroxyl group to an isopropyl group is predicted to have one of two effects. Either the character of the protein surface will be changed from polar to hydrophobic at that location, or, by altering the amphipathic nature of that helix, the mutation could affect the helix packing and positioning, resulting in a larger structural change such as rotation of the helix. Either of these two effects could explain the functional consequence of the variant.

### F559I

F559 is a bulky aromatic residue found on the interior of the protein model, within 9 Å of the predicted DNA-binding interface (Fig. 3b). Replacement of the large volume of the phenylalanine side chain with the smaller volume of isoleucine could alter the geometry of the DNA-binding cavity sufficiently to disrupt that interaction. Notably, while F559 is in the second shell of residues responsible for DNA contact, it is predicted to be directly adjacent to two first-shell residues, E591 and A621, which have been previously reported as disease-associated [28].

### S688C

S688 is located on a buried helix one turn (5.9 Å) away from disease-associated residue R684. The mutation of serine to cysteine does not result in major changes in bulk, branching, charge, or hydrophobicity. However, the presence of the sulfhydryl group in the cysteine could potentially promote misfolding and aggregation upon incorrect formation of disulfide bonds, if exposed to oxidation.

### D719G

D719 is located on a surface-exposed helix near the pathogenic cluster (Fig. 3c). Replacing the large charged aspartate sidechain with the single hydrogen of a glycine removes a bulky charge from the protein surface and likely disrupts the helix in that region.

### T55S

T55 is a polar residue predicted to lie at the interface between alpha helices 1 and 2 (Fig. 3d). Relative to the other segregating variants, T55S is distal to the pathogenic cluster and is relatively equidistant to pathogenic and neutral variation. Both threonine and serine are unusual residues to find in a helix-helix interface, and suggest that this position may be functionally important. Replacement of a threonine sidechain with that of serine does not alter the hydroxyl character of the residue, though it reduces the steric bulk by one methyl group. This is not a major volumetric change, but the removal of a beta-branching amino acid could affect inter-helical packing. This steric change could result in a relative repacking of the helix-helix interface, or could change the strength of interaction between the helices. Another mutation in this helix (K48R) has been shown to abolish ATPase activity when mutated to arginine [57], though this mutation is also physically closer to the ATP-binding cleft. Although T55 is evolutionarily conserved, SIFT and PolyPhen2 each confidently predict the serine substitution to be benign. Ultimately, there is no obvious structural basis for the pathogenicity of T55S and its distance from the pathogenic cluster suggests that any functional effects are likely impacting alternative mechanisms.

In comparison to general pathogenicity-prediction algorithms, this approach makes use of dense population and disease-association data for variants specifically in *RTEL1* using conservative assumptions of pathogenicity. Consequently, the availability of well-characterized pathogenic and neutral variants in the protein-of-interest is essential. The incorporation of variants and mutagenesis data from functional homologs may help to overcome this limitation. For example, the spatial distribution of disease-causing missense variants in RTEL1 suggests that the ATP-binding cleft between helicase domains I and II and the DNA-binding pore along helicase domain II are functionally critical regions of RTEL1. This finding is consistent with observed patterns of missense variants associated with *Xeroderma pigmentosum* (XP) in the homologous protein XPD [46]. While variants in XPD have different phenotypic presentations than those in RTEL1, the overlapping regions of pathogenicity suggest similar functional effects, with higher-order phenotypes driven by cellular context or unique functional domains (e.g. RTEL1 harmonin-N-like domains). This hypothesis is supported by the significant correlation between RTEL1-derived pathogenic proximity scores and reduced ATPase activity in XPD. This algorithm can be iteratively enhanced as additional disease-associated variants and primary/homologous mutagenesis data become available.

Assigning pathogenicity to missense variants in RTEL1 presents unique challenges. An ideal biomarker/assay of RTEL1 activity has not been defined, and likely differs based on the specific mutation. Short PBMC telomeres appear to be a common feature associated with RTEL1 mutations, but it is not yet clear whether this is a uniform feature; telomere length in RTEL null mouse embryonic stem cells appears stable [58], so preserved telomere length alone may not sufficiently exclude deleterious function of RTEL1 variants. In light of these complexities, for algorithm training, we conservatively defined variants as pathogenic only if they had been reported to be associated with severe pediatric disease in a recessive genetic model. For testing on novel VUS, we considered segregation with disease and telomere length in defining likely pathogenic variants. Our method classified five of the six VUS that co-segregated with FIP as pathogenic, but it also misclassified three VUS. This may demonstrate a lack of specificity when considering only the location of variants within protein structure. Spatial information demonstrates predictive potential, but it does not directly capture the impact of specific amino acid substitutions, evolutionary conservation, or biochemical information critical for interpretation. However, the specificity of our approach is comparable with other prediction methods, nearly all of which also misclassified the three VUS. It is also possible that these “misclassified” variants do adversely affect RTEL1 function without leading to a direct effect on telomere length [58]; comprehensive evaluation of these variants and others over-time should lend more clarity. At present, technical issues have limited the ability to perform in-vitro studies in overexpression systems [58]. In addition, it is possible that more than one dominant risk mutation could be found in a family; in this case, lack of co-segregation would not exclude a pathogenic effect.

We have focused our analysis on disease-causing variants in *RTEL1* with a particular interest in predicting variants that increase risk for FIP. However, the methodology is dependent only on the availability of protein structural information (whether experimentally derived or computationally predicted) and the assumption that disease-causing variants are spatially clustered within the protein structure. The tendency for cancer-associated somatic mutations to form spatial clusters in protein sequence and structure is well established [59], and initial evidence for spatial clustering has likewise been observed for germline disease-causing variants [60, 61]. Thus, the methodology proposed here will likely be broadly useful in the identification of disease regions of interest within protein structure and variant pathogenicity prediction.

## Conclusions

Our results demonstrate that considering the 3D spatial landscape of missense variation in RTEL1 has the potential to improve pathogenicity prediction and identify functional regions of protein structure important to the development of disease. We implicate the ATP-binding cleft between helicase domains I and II as well as the DNA-binding pore along helicase domain II as functional regions of RTEL1 contributing to the development of FIP. The similar distributions of disease-associated variants and a significant correlation with ATPase activity in the homologous protein XPD support this finding and suggest that including additional variants from homologous proteins may improve predictive power and discover shared biochemical etiology. More generally, we propose incorporating the spatial distributions of known pathogenic and neutral variation into pathogenicity prediction methods to complement existing predictive features, particularly for proteins in which pathogenic variants appear to form clusters within protein structure. Ultimately, the use of this information has the potential to enhance the utility of genetic data in elucidating the etiology of FIP and other heritable diseases.

## Additional files

Additional file 1: Supplementary Methods, Results, Figures, and Tables. (DOCX 849 kb)

Additional file 2: Computational homology model of the protein structure of RTEL1. (PDB 1274 kb)

Additional file 3: Mutagenesis data and RTEL1-mapping for Fan et al. and Kuper et al. XPD variants. (XLSX 13 kb)
