# Supplementary material for: Three-dimensional spatial analysis of missense variants in RTEL1 identifies pathogenic variants in patients with Familial Interstitial Pneumonia
Source: BMC Bioinformatics. 2018 Jan 23;19:18. doi: 10.1186/s12859-018-2010-z (PMC5781290; doi:10.1186/s12859-018-2010-z)
Supplement: Supplementary file 1 — Supplementary Methods, Results, Figures, and Tables. (DOCX 849 kb) [file 12859_2018_2010_MOESM1_ESM.docx]

**Supplemental Methods**

***Pathogenicity prediction methods***

ConSurf [1] calculates the relative evolutionary conservation of each amino acid within a protein sequence, with scores ranging from –1.5 (most conserved) to 1.5 (least conserved). Variants were ranked by increasing ConSurf score; amino acid substitutions at the most conserved residues were predicted to be deleterious, and at the least conserved residues to be neutral. SIFT [2] and PolyPhen2 [3] are machine learning classifiers designed to predict the impact of amino acid substitutions using a combination of sequence- and/or structure-derived features. SIFT uses multiple sequence alignments of closely-related homologs to calculate the probabilities of amino acid substitutions. Substitutions with low likelihoods are predicted to be deleterious while those with moderate or high likelihoods are predicted to be benign. PolyPhen2 provides posterior probability estimates of pathogenicity ranging from 0 (benign) to 1 (damaging). SIFT and PolyPhen2 scores were calculated by the Ensembl Variant Effect Predictor [4].

**Supplemental Results**

***Quantitative spatial analysis of variants in the RTEL1 C-terminal model***

The C-terminal model of RTEL1 (residues 881-1151) contained only four known pathogenic variants, for which we observed no spatial clustering (Figure S3a). As described for the N-terminal model, we used leave-one-out cross validation to evaluate the predictive performance of pathogenic proximity in the C-terminal model (Table S2, Figure S3b). We obtained a lower ROC AUC of 0.70 than in the N-terminal analysis, indicating reduced predictive benefit in the absence of dense pathogenic clustering. However, neither VUS in the C-terminal model (F1110L and P1107L) segregated with disease and both were correctly predicted to be neutral by pathogenic proximity.

**Supplementary Tables**

| **Pos** | **Ref** | **Alt** | **Type** | **PPH2** | **SIFT** | **ConSurf** | **PathProx** |
| --- | --- | --- | --- | --- | --- | --- | --- |
| 512 | W | C | Unknown | 0.17 | 0.48 | 0.31 | 0.47 |
| 55 | T | S | Seg | 0.00 | 1.00 | -0.56 | -0.02 |
| 516 | V | L | Seg | 0.05 | 0.62 | -0.15 | 0.41 |
| 540 | S | A | Seg | 0.57 | 0.09 | -0.80 | 0.21 |
| 559 | F | I | Seg | 1.00 | 0.00 | -1.11 | 0.44 |
| 688 | S | C | Seg | 0.91 | 0.14 | -0.62 | 0.27 |
| 719 | D | G | Seg | 0.03 | 0.22 | 0.21 | 0.05 |
| 161 | H | Q | NonSeg | 0.40 | 0.16 | -0.35 | -0.13 |
| 397 | Q | E | NonSeg | 0.08 | 0.20 | 0.40 | -0.09 |
| 528 | A | E | Unknown | 0.62 | 0.05 | -0.75 | 0.08 |
| 574 | R | W | NonSeg | 0.95 | 0.00 | -0.53 | 0.07 |
| 251 | E | K | Pathogenic | 1.00 | 0.00 | -1.13 | 0.08 |
| 492 | M | I | Pathogenic | 0.07 | 0.02 | -0.67 | 0.02 |
| 591 | E | D | Pathogenic | 1.00 | 0.01 | -1.03 | -0.01 |
| 699 | I | M | Pathogenic | 1.00 | 0.00 | -1.13 | 0.22 |
| 710 | L | R | Pathogenic | 1.00 | 0.00 | -0.91 | 0.28 |
| 739 | G | V | Pathogenic | 1.00 | 0.00 | -0.90 | 0.22 |
| 745 | V | M | Pathogenic | 0.24 | 0.06 | -0.58 | 0.30 |
| 74 | E | K | Neutral | 0.05 | 0.50 | 0.71 | -0.05 |
| 84 | R | P | Neutral | 0.01 | 0.10 | 1.47 | -0.03 |
| 87 | S | T | Neutral | 0.02 | 0.14 | 1.33 | -0.07 |
| 112 | A | T | Neutral | 0.97 | 0.00 | -0.64 | 0.10 |
| 113 | S | P | Neutral | 1.00 | 0.00 | -1.01 | 0.08 |
| 124 | N | S | Neutral | 0.00 | 0.43 | 0.65 | 0.00 |
| 135 | V | G | Neutral | 0.29 | 0.12 | 0.04 | -0.10 |
| 167 | V | M | Neutral | 0.95 | 0.01 | -0.50 | -0.10 |
| 178 | V | A | Neutral | 0.37 | 0.00 | -0.41 | -0.11 |
| 178 | V | I | Neutral | 0.11 | 0.14 | -0.41 | -0.11 |
| 209 | Y | H | Neutral | 1.00 | 0.00 | -0.97 | -0.16 |
| 235 | R | L | Neutral | 1.00 | 0.00 | -1.00 | -0.01 |
| 268 | T | A | Neutral | 0.40 | 0.09 | -0.10 | -0.10 |
| 278 | V | I | Neutral | 0.00 | 0.13 | -0.14 | -0.11 |
| 287 | T | I | Neutral | 0.03 | 0.19 | 0.91 | -0.06 |
| 320 | M | T | Neutral | 0.01 | 0.56 | 0.43 | -0.10 |
| 328 | A | G | Neutral | 0.02 | 0.00 | -0.31 | -0.08 |
| 340 | G | S | Neutral | 0.90 | 0.07 | -0.89 | -0.02 |
| 354 | E | K | Neutral | 0.00 | 1.00 | 0.46 | -0.08 |
| 379 | R | C | Neutral | 0.34 | 0.02 | 0.71 | -0.06 |
| 397 | Q | E | Neutral | 0.08 | 0.20 | 0.40 | -0.05 |
| 434 | T | M | Neutral | 0.30 | 0.12 | 0.70 | 0.01 |
| 483 | A | T | Neutral | 0.49 | 0.01 | -1.00 | 0.25 |
| 576 | R | H | Neutral | 0.01 | 0.47 | 1.32 | 0.00 |
| 600 | E | K | Neutral | 0.78 | 0.45 | 0.73 | 0.06 |
| 621 | A | V | Neutral | 1.00 | 0.00 | -1.02 | 0.32 |
| 636 | T | M | Neutral | 0.04 | 0.15 | 1.40 | 0.19 |
| 684 | R | Q | Neutral | 0.02 | 0.69 | 0.19 | 0.09 |
| 753 | E | D | Neutral | 0.01 | 0.17 | 0.66 | 0.19 |

Table S1: All N-terminal missense variants and pathogenicity predictions.

| **Pos** | **Ref** | **Alt** | **Type** | **PPH2** | **SIFT** | **ConSurf** | **PathProx** |
| --- | --- | --- | --- | --- | --- | --- | --- |
| 1107 | P | L | NonSeg | 0.63 | 0.01 |  | -0.13 |
| 1110 | F | L | NonSeg | 0.00 | 1.00 |  | -0.17 |
| 897 | K | E | Pathogenic | 0.72 | 0.12 | 0.76 | -0.08 |
| 957 | R | W | Pathogenic | 0.99 | 0.00 | -0.31 | 0.13 |
| 964 | F | L | Pathogenic | 0.44 | 0.07 | -0.31 | 0.03 |
| 1042 | Q | H | Pathogenic | 0.00 |  |  | -0.02 |
| 884 | P | L | Neutral | 0.00 | 0.13 | -0.50 | -0.02 |
| 888 | V | L | Neutral | 0.03 | 0.31 | -0.50 | 0.13 |
| 893 | T | M | Neutral | 0.00 | 0.13 | -0.46 | 0.23 |
| 898 | L | F | Neutral | 0.01 | 0.08 | -0.55 | 0.18 |
| 929 | A | T | Neutral | 0.05 | 0.12 | -0.31 | 0.52 |
| 933 | A | V | Neutral | 0.09 | 0.26 | -0.31 | 0.40 |
| 937 | P | L | Neutral | 0.00 | 0.30 | -0.31 | 0.11 |
| 941 | E | D | Neutral | 0.36 | 0.15 | -0.31 | -0.08 |
| 942 | D | N | Neutral | 0.09 | 0.26 | -0.31 | -0.09 |
| 966 | E | D | Neutral | 0.01 | 0.58 | -0.31 | 0.13 |
| 968 | C | S | Neutral | 0.87 | 0.00 | -0.31 | 0.11 |
| 972 | T | I | Neutral | 0.05 | 0.04 | -0.31 | -0.10 |
| 978 | Y | H | Neutral | 0.02 | 0.01 | -0.31 | -0.12 |
| 979 | R | Q | Neutral | 0.00 | 0.93 | -0.31 | -0.20 |
| 979 | R | W | Neutral | 0.39 | 0.01 | -0.31 | -0.20 |
| 981 | E | K | Neutral | 0.01 | 0.33 | -0.31 | -0.18 |
| 982 | H | R | Neutral | 0.31 | 0.01 | -0.31 | -0.25 |
| 989 | R | P | Neutral | 0.21 | 0.25 | -0.31 | -0.19 |
| 989 | R | W | Neutral | 0.00 | 0.02 | -0.31 | -0.19 |
| 992 | P | L | Neutral | 0.00 | 0.18 | -0.31 | -0.15 |
| 1000 | T | M | Neutral | 0.00 | 0.11 | -0.31 | -0.15 |
| 1008 | V | M | Neutral | 0.11 | 0.08 | -0.31 | -0.23 |
| 1013 | A | V | Neutral | 0.00 | 0.92 | -0.31 | -0.17 |
| 1017 | D | N | Neutral | 0.03 | 0.43 | -0.31 | -0.19 |
| 1019 | Q | R | Neutral | 0.00 | 0.44 | -0.31 | -0.23 |
| 1022 | L | V | Neutral | 0.79 | 0.00 | -0.31 | -0.20 |
| 1027 | P | L | Neutral | 0.01 | 0.03 | -0.31 | -0.24 |
| 1034 | P | H | Neutral | 0.34 | 0.29 | -0.31 | 0.05 |
| 1056 | G | D | Neutral | 0.01 | 0.62 |  | -0.10 |
| 1059 | A | T | Neutral | 0.01 | 0.28 |  | 0.25 |
| 1060 | V | M | Neutral | 0.37 | 0.12 |  | 0.14 |
| 1068 | R | H | Neutral | 0.01 | 0.40 |  | 0.17 |
| 1074 | A | V | Neutral | 0.17 | 0.05 |  | 0.08 |
| 1084 | T | I | Neutral | 0.05 | 0.16 |  | -0.19 |
| 1091 | D | N | Neutral | 0.01 | 0.26 |  | -0.17 |
| 1099 | L | S | Neutral | 0.08 | 0.00 |  | -0.17 |
| 1104 | T | I | Neutral | 1.00 | 0.00 |  | -0.10 |
| 1107 | P | R | Neutral | 0.64 | 0.12 |  | -0.11 |
| 1115 | R | G | Neutral | 0.00 | 0.19 |  | -0.21 |
| 1137 | G | S | Neutral | 1.00 | 0.00 |  | -0.11 |
| 1141 | P | L | Neutral | 0.00 | 0.32 |  | -0.11 |
| 1145 | P | S | Neutral | 0.00 | 0.73 |  | -0.14 |
| 1148 | P | L | Neutral | 0.01 | 0.13 |  | -0.07 |

Table S2: All C-terminal missense variants and pathogenicity predictions.

**Supplementary Figures**


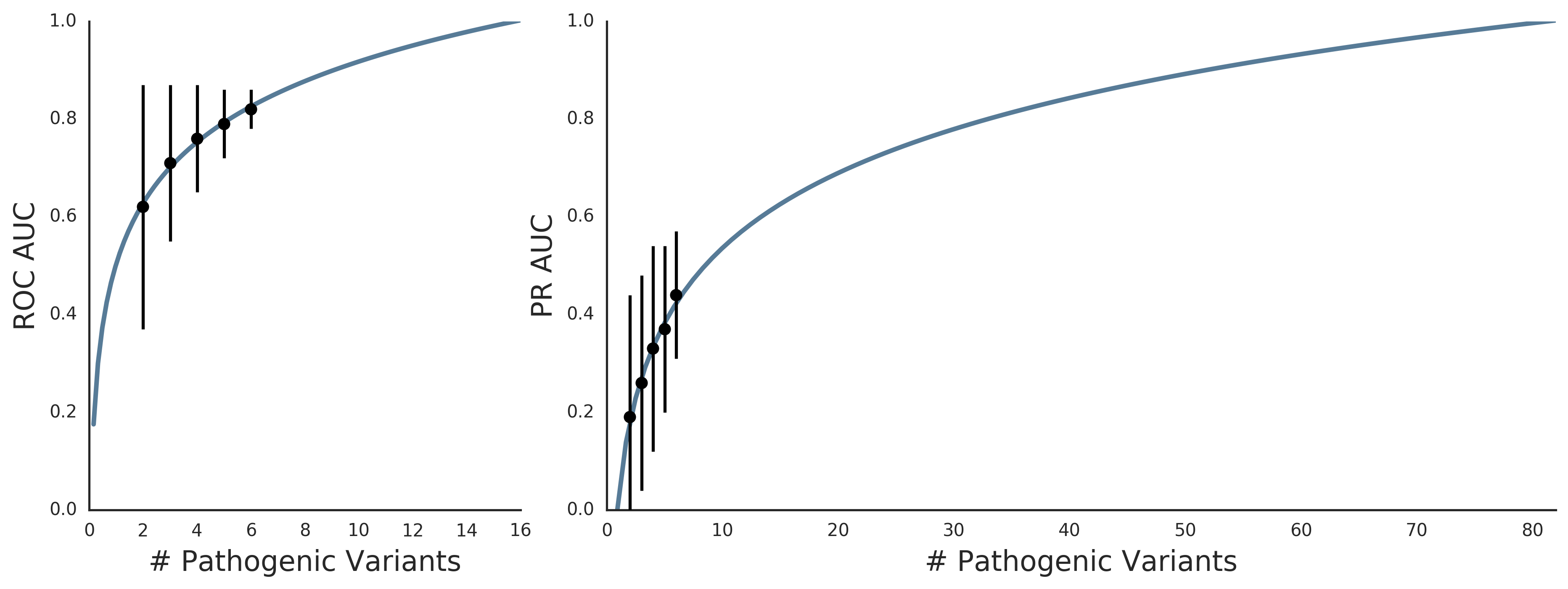


Figure S1: Estimation of the sensitivity of pathogenic-proximity-based prediction to the number of known pathogenic variants. ROC AUC (a) and PR AUC (b) were computed based on all subsets of the seven known pathogenic variants in RTEL1’s N-terminal domain. The black dots represent the mean performance across all subsets of each size (subsets of 2 to 6 variants) and the bars represent the standard deviation. The blue curve was fit to the log-AUC using linear regression.


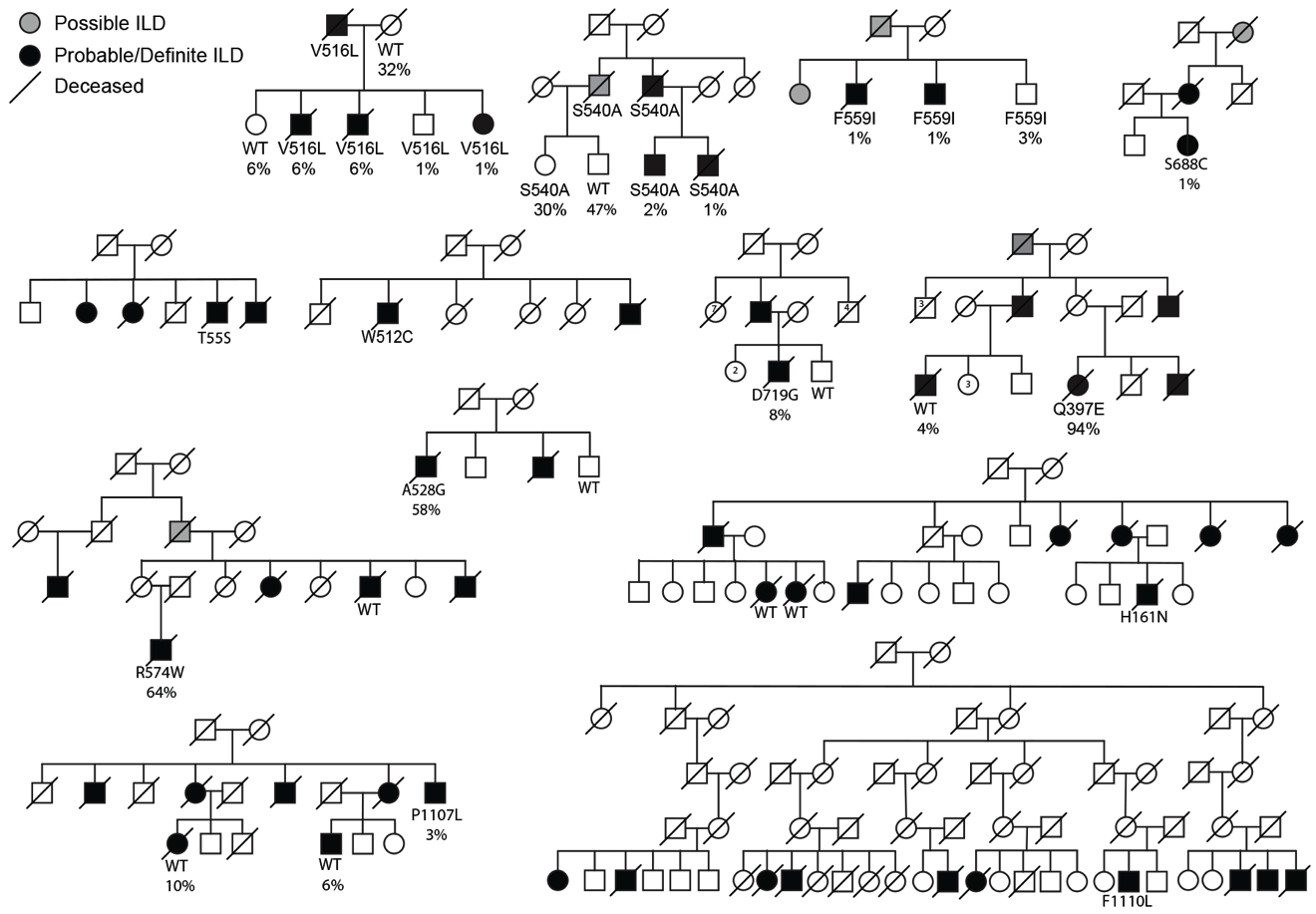


Figure S2: Genotyping of 373 FIP patients identified 13 missense variants of unknown significance (VUS) in RTEL1. Analysis of pedigrees of FIP patients demonstrated that seven VUS segregate with disease. Telomere percentages are provided below each mutation when available.


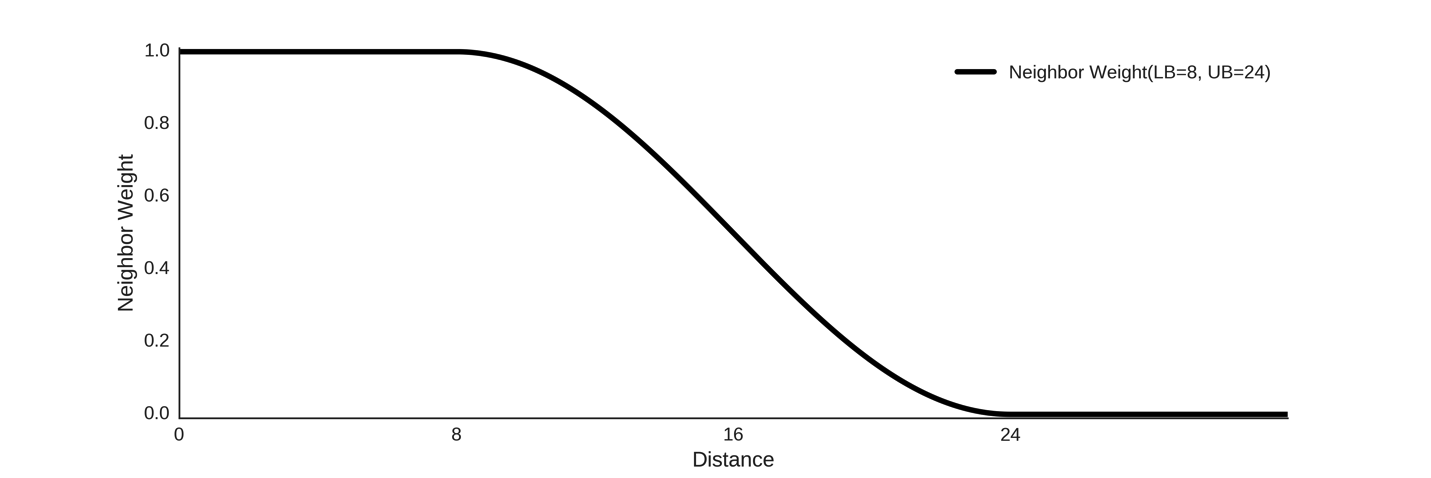


Figure S3: Neighbor Weight as a function of distance using a lower bound of 8Å and an upper bound of 24Å.


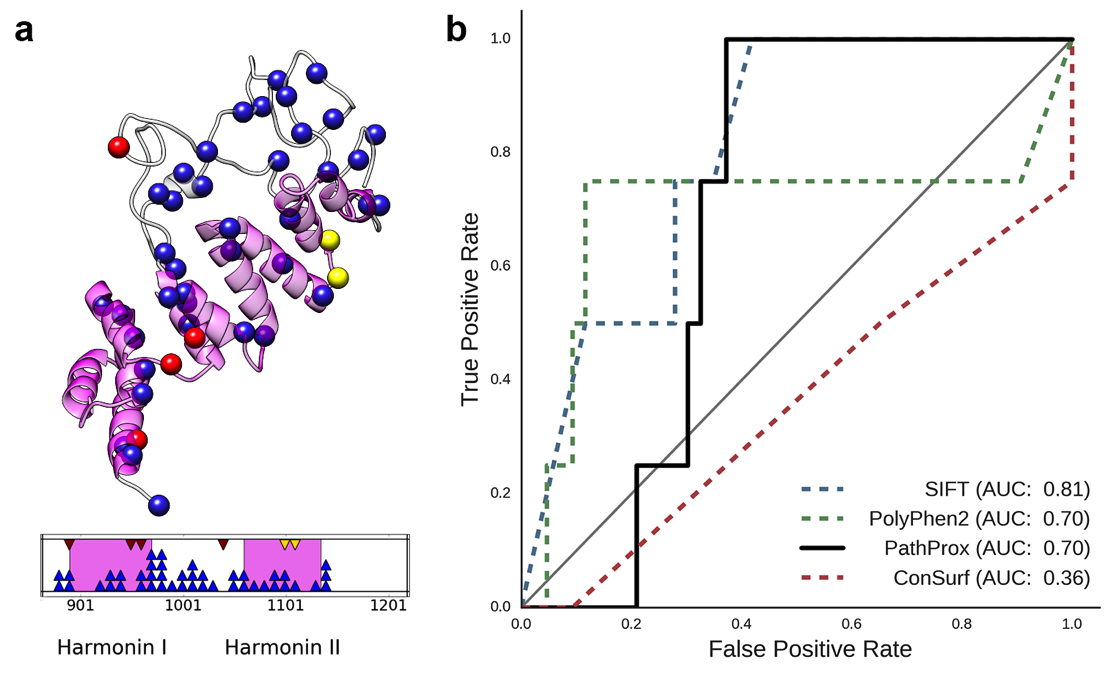


**Figure S4:** (a) The locations of known ClinVar pathogenic (red), putatively neutral 1000 Genomes (blue), and new candidate FIP (yellow) missense variants in the RTEL1 C-terminal structural model. (b) Receiver operating characteristic (ROC) curves for variants in the C-terminal model of RTEL1. ConSurf did not provide conservation scores for most residues in the C-terminal model. Only four pathogenic variants were present in the C-terminal model and predictive performance was notably worse than in the larger N-terminal model.


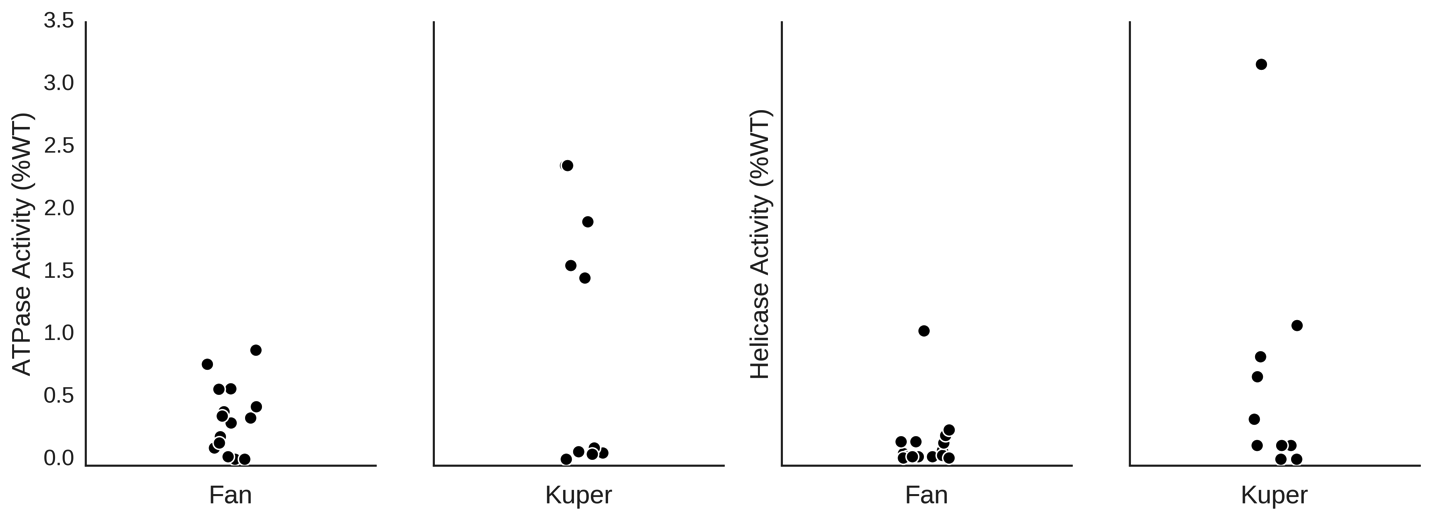


**Figure S5:** ATPase and helicase reported activity (as percentage of wild type) for missense mutations in saXPD and taXPD.

**References**

1. Goldenberg O, Erez E, Nimrod G, Ben-Tal N. The ConSurf-DB: Pre-calculated evolutionary conservation profiles of protein structures. Nucleic Acids Res. 2009;37:323–7.

2. Ng PC, Henikoff S. SIFT: Predicting amino acid changes that affect protein function. Nucleic Acids Res. 2003;31:3812–4.

3. Adzhubei IA, Schmidt S, Peshkin L, Ramensky VE, Gerasimova A, Bork P, et al. A method and server for predicting damaging missense mutations. Nat. Methods. Nature Publishing Group; 2010;7:248–9.

4. McLaren W, Pritchard B, Rios D, Chen Y, Flicek P, Cunningham F. Deriving the consequences of genomic variants with the Ensembl API and SNP Effect Predictor. Bioinformatics. 2010;26:2069–70.
